# Supplementary material for: NASICON-type air-stable and all-climate cathode for sodium-ion batteries with low cost and high-power density
Source: Nat Commun. 2019 Apr 1;10:1480. doi: 10.1038/s41467-019-09170-5 (PMC6443767; doi:10.1038/s41467-019-09170-5)
Supplement: Supplementary file 1 — Supplementary Information [file 41467_2019_9170_MOESM1_ESM.pdf]

# Supplementary Information

## New NASICON-Type Air Stable and All-Climate Cathode for Sodium-Ion Batteries with Low-Cost and High-Power Density

**Chen et al.**

## Supplementary Figures

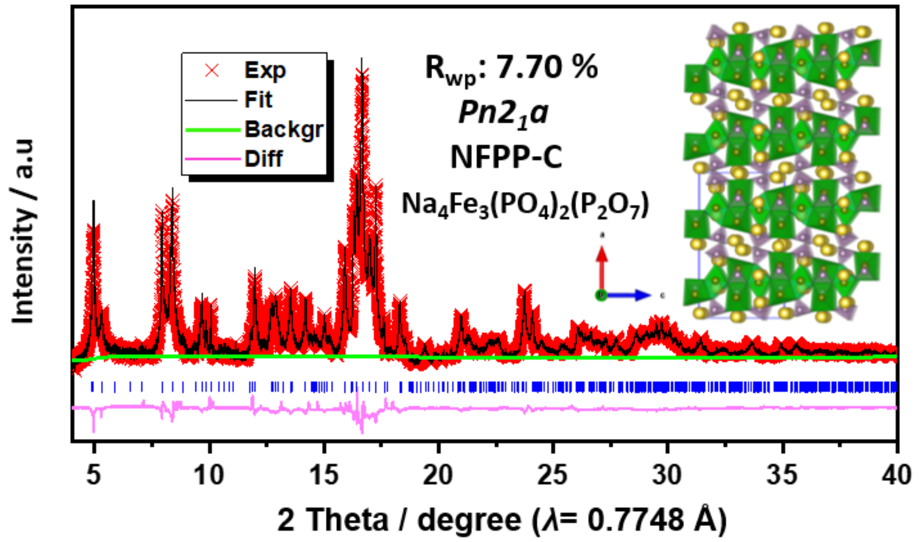


**Supplementary Figure 1** Rietveld refinements of NFPP-C samples. A Schematic representation of the refinement results is presented as inset.

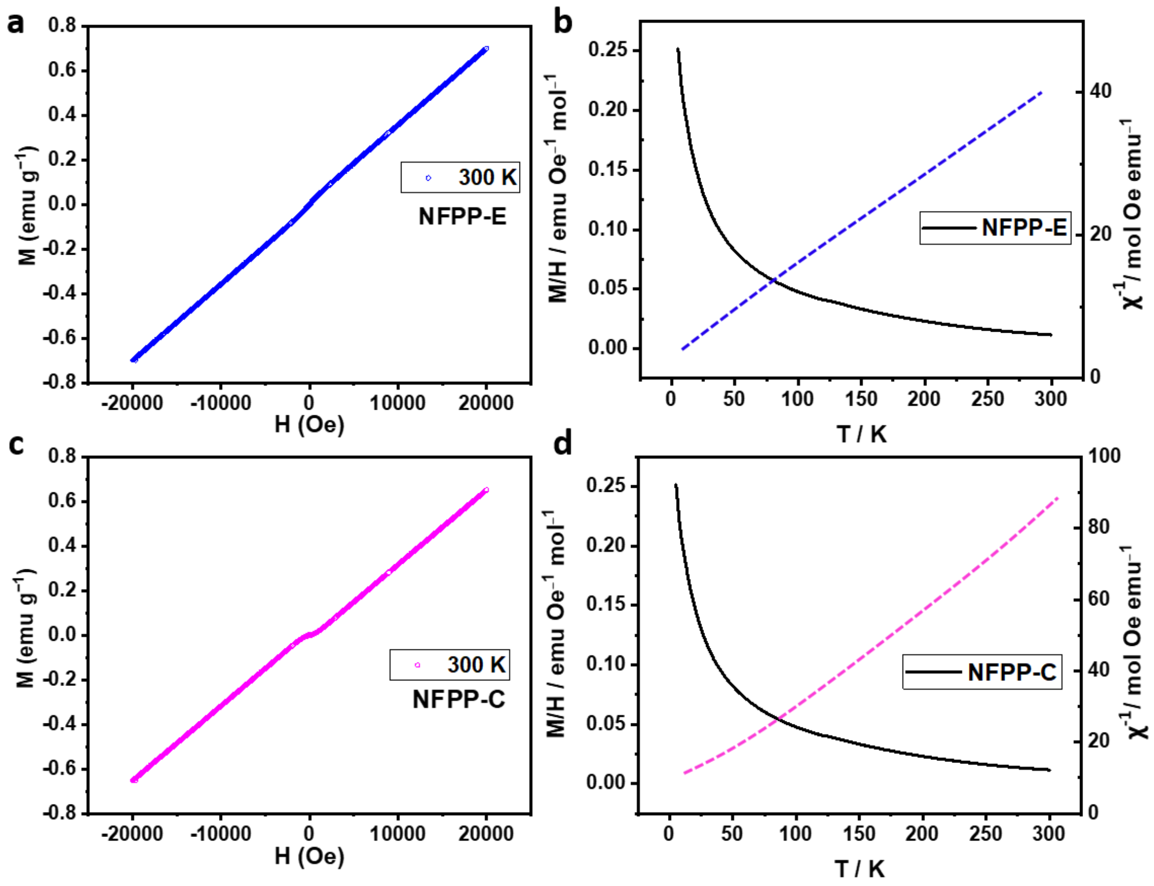


**Supplementary Figure 2** Magnetization properties. Magnetization (*M*-*H*) curves of (a) NFPP-E and (c) NFPP-C samples at 300 K. Temperature dependence of magnetic susceptibility (*M*-*T*) (black line) curves of (b) NFPP-E and (d) NFPP-C in an applied field of 1000 Oe. The dashed blue line in (b) and the dashed magenta line in (d) are the fitted inverse susceptibility results for NFPP-E and NFPP-C, respectively.

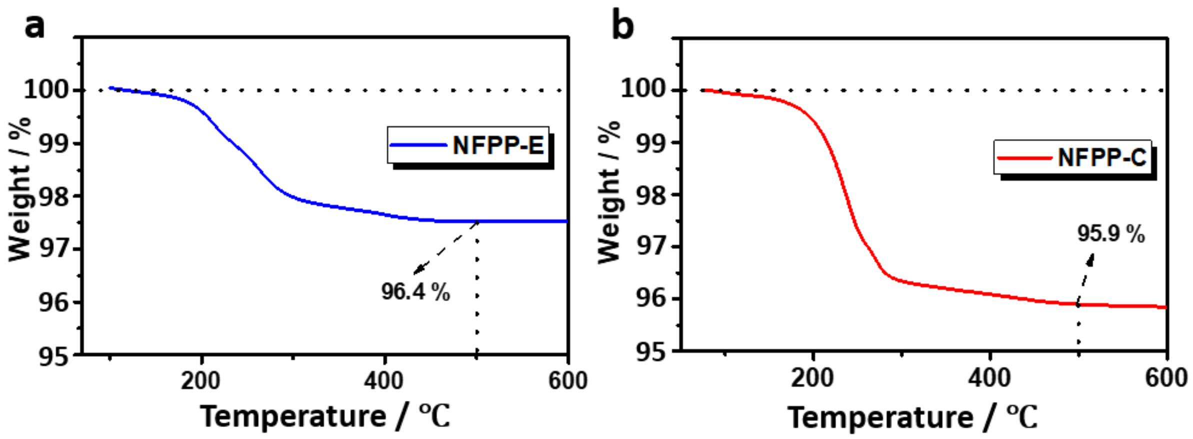


**Supplementary Figure 3** Thermogravimetric analysis. Thermogravimetric analysis in air atmosphere of the (a) NFPP-E and (b) NFPP-C samples, respectively.

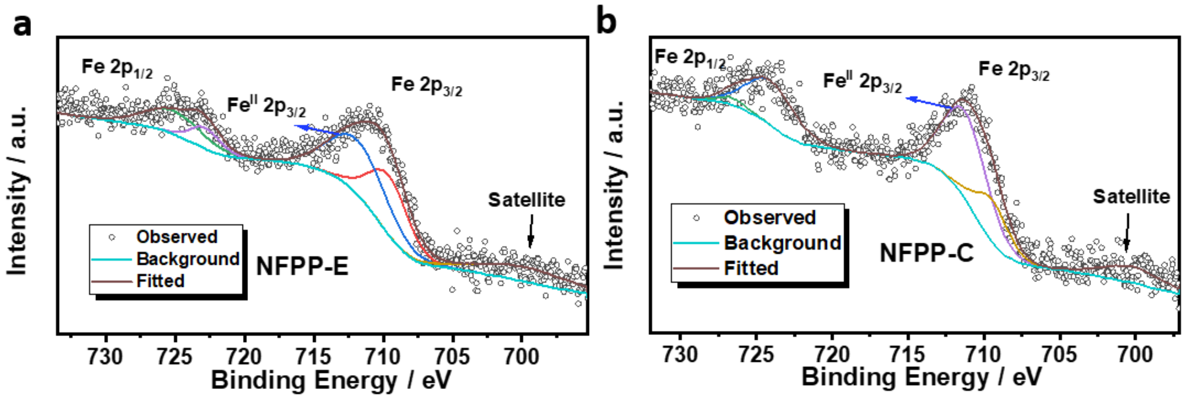


**Supplementary Figure 4** Surface information. X-ray photoelectron spectroscopy (XPS) results on Fe for the (a) NFPP-E and (b) NFPP-C samples.

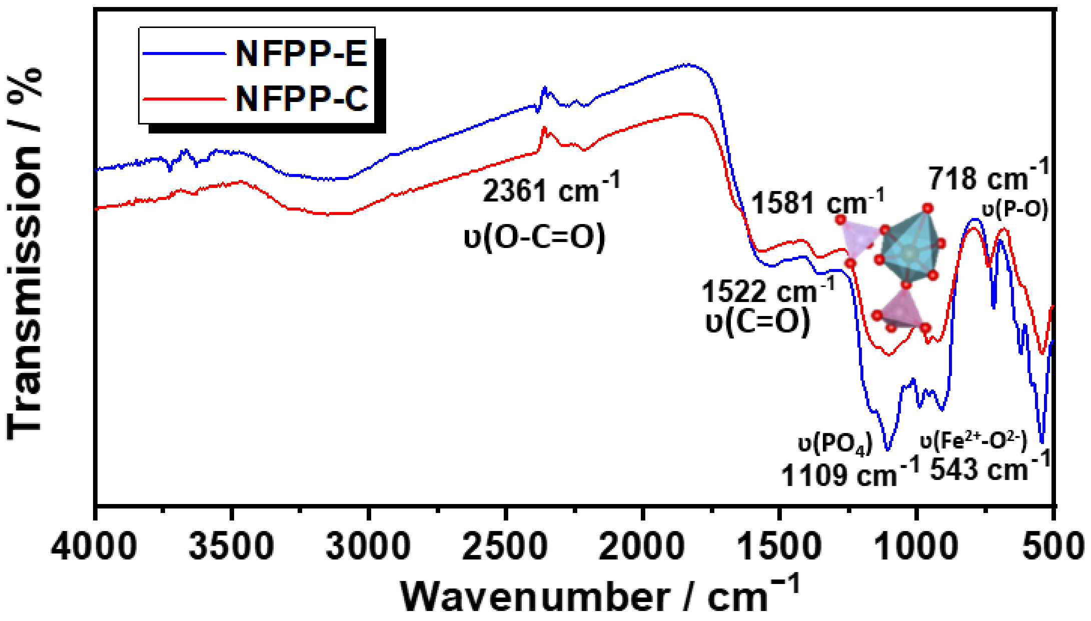


**Supplementary Figure 5** Phase characterization. Comparison of FT-IR spectra of NFPP-E and NFPP-C samples.

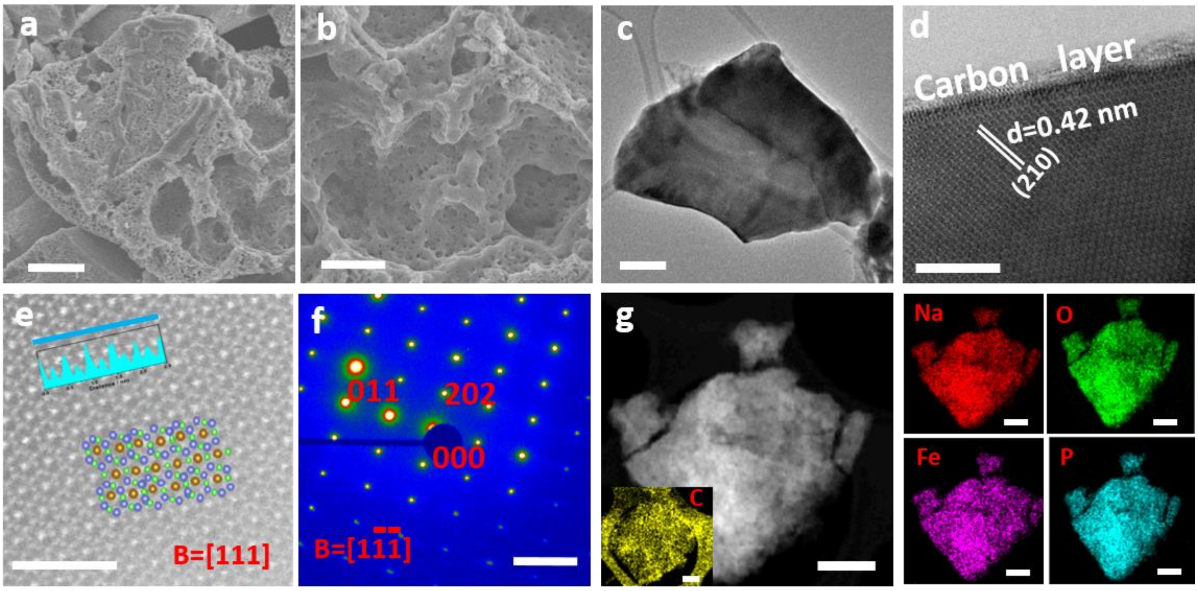


**Supplementary Figure 6** Morphology characterizations of NFPP-C samples. (a) and (b) SEM images of NFPP-C at different magnifications. Transmission electron microscope (TEM) image of (c) N NFPP-C and (d) Bright Field (BF) image of NFPP-C with carbon layers., respectively. HAADF image from aberration-corrected STEM and the crystal structure of (e) NFPP-E viewed from the [010] direction. The insets are the corresponding signal response along the selected lines. (f) SAED pattern of NFPP-E. (g) The STEM-EDS mapping results with selected elements of NFPP-E. Scale bars: 500 nm (a); 100 nm (b); 200 nm (c); 5 nm (d); 2 nm (e); 2 1/nm (f); 200 nm (g).

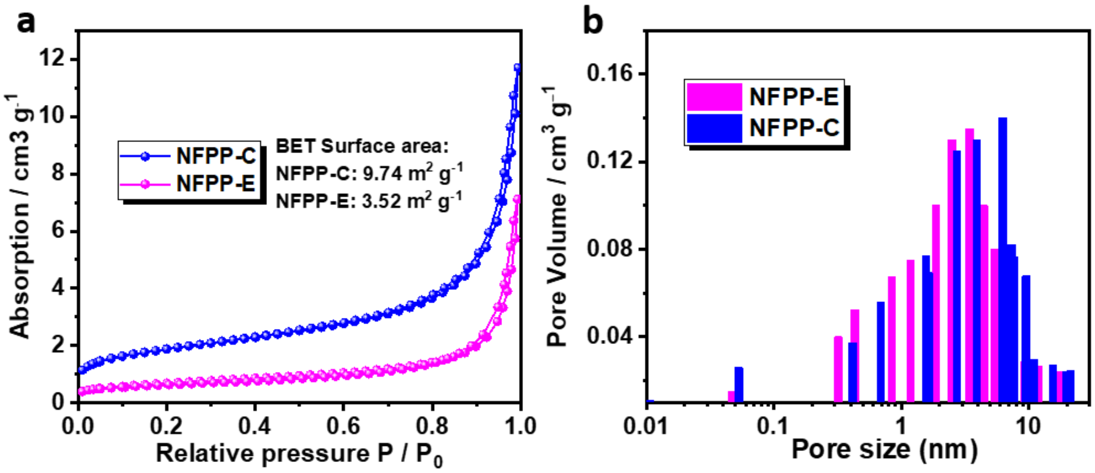


**Supplementary Figure 7** Surface absorption and pore size. (a) BET testing results for both samples. (b) Pore size distributions of both samples.

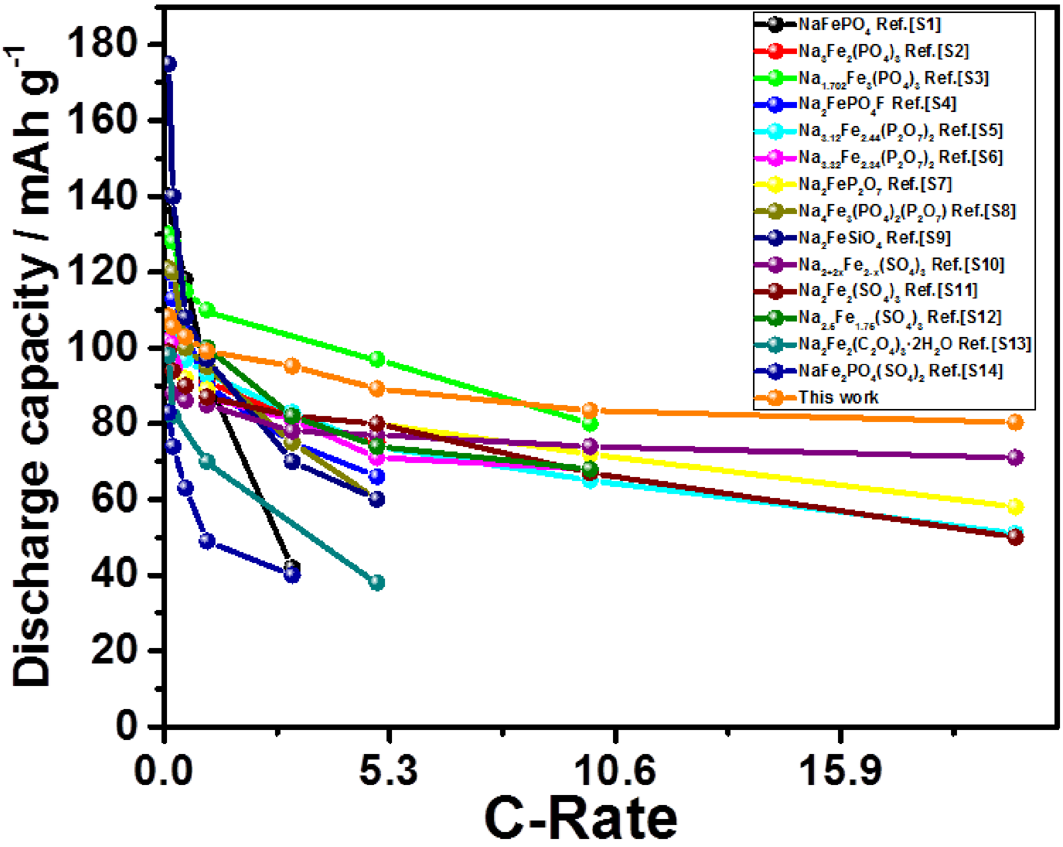


**Supplementary Figure 8** C-rate comparison of recently published iron-based polyanionic materials (Supplementary References 1-14).

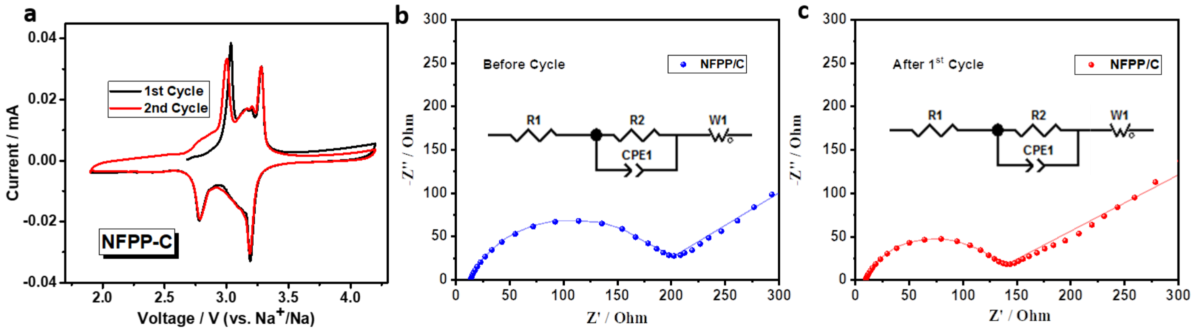


**Supplementary Figure 9** Electrochemical properties of NFPP-C sample. (a) CV curves of NFPP-C electrode for the first two cycles (scan rate 0.05 mV $s^{-1}$). EIS spectra of NFPP-E electrode (b) before cycling and (c) after the first cycle. The insets are the equivalent circuits used for interpreting the data. CPE: constant phase element, W: Warburg impedance.

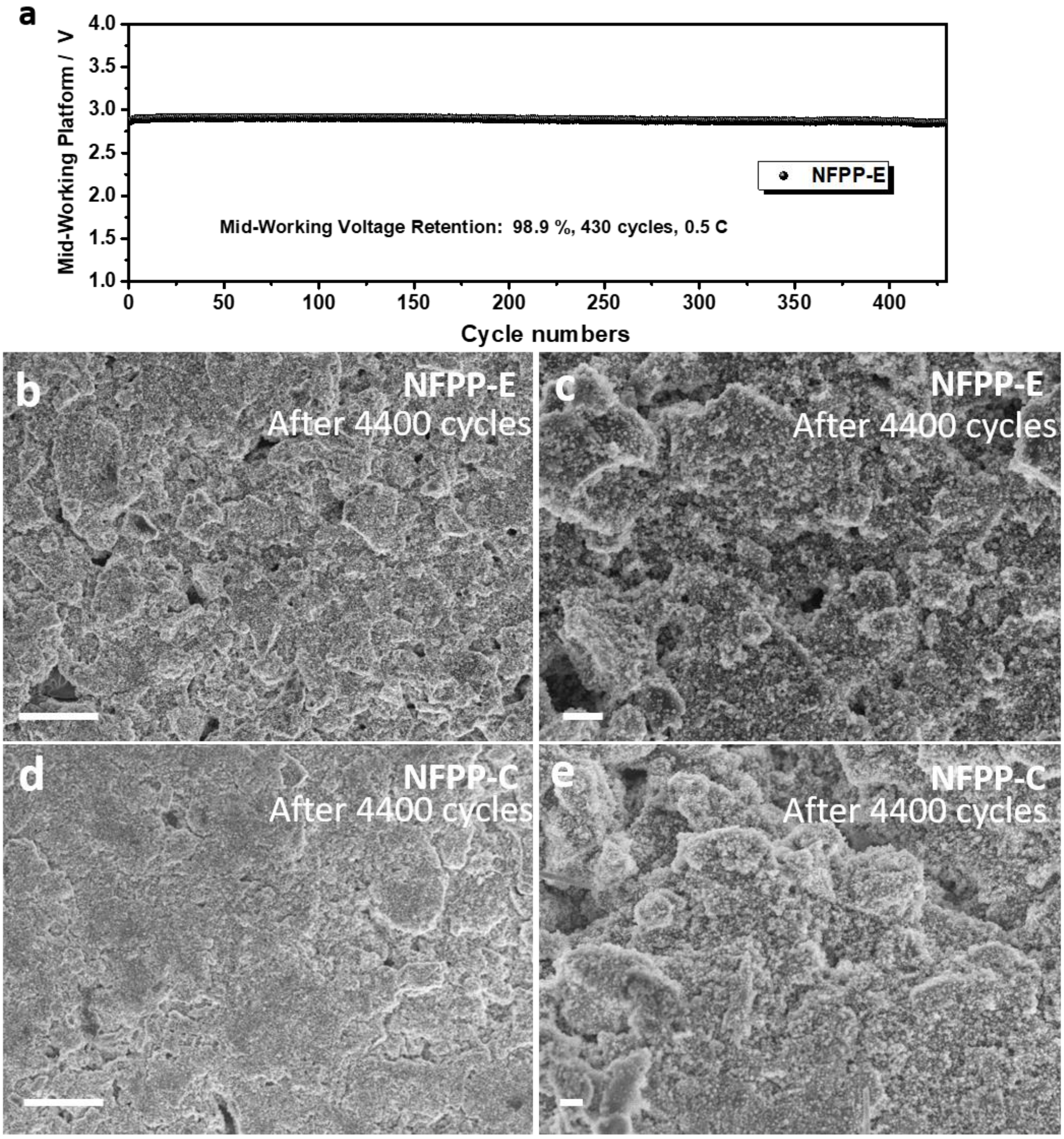


**Supplementary Figure 10** Working potential retention and SEM images of cycled electrodes. (a) Mid-working platform retention of NFPP-E electrode over 430 cycles at 0.5 Cs. (b) and (c) SEM images of NFPP-E after 4400 cycles. (d) and (e) SEM images of NFPP-C after 4400 cycles. Scale bars: 10 μm (b); 2 μm (c); 10 μm (d); 1 μm (e).

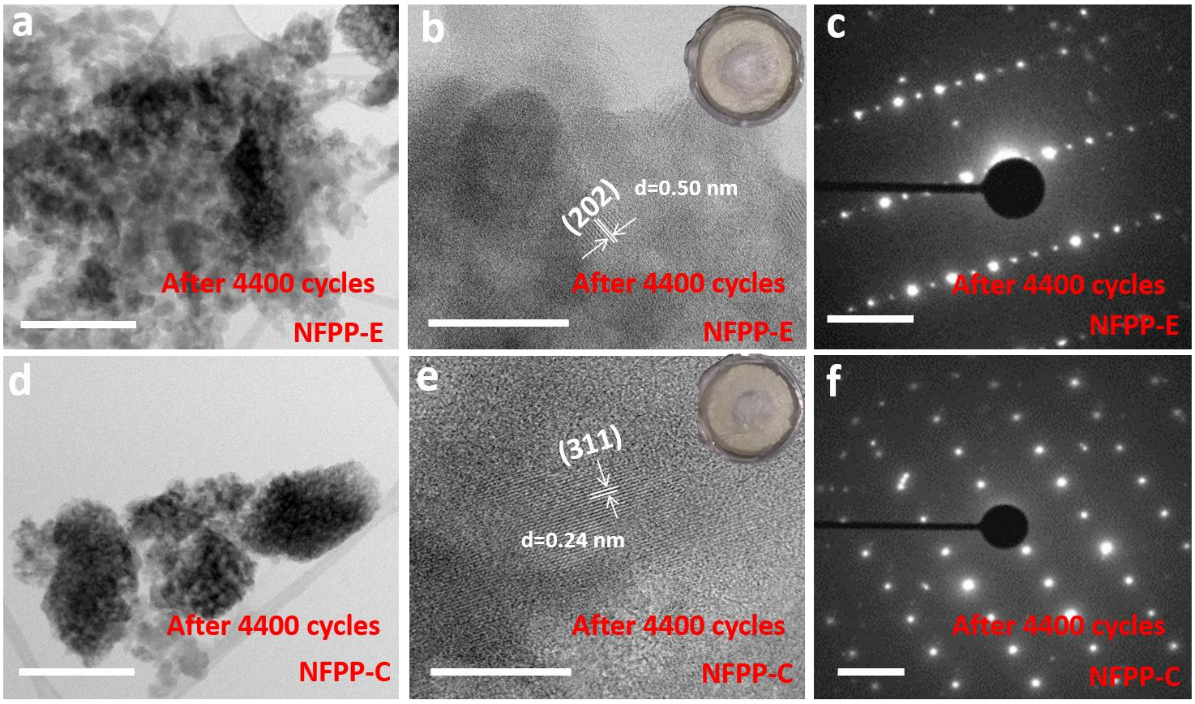


**Supplementary Figure 11** TEM images of cycled electrodes. TEM and HRTEM images of (a) and (b) NFPP-E electrode and (d) and (e) NFPP-C electrode after 4400 cycles. The insets in (b) and (e) are images of the glass fiber separators after disassembly. (c) and (f) present the corresponding SAED patterns of both electrodes. Scale bars: 500 nm (a); 20 nm (b); 2 1/nm (c); 500 nm (d); 10 nm (e); 2 1/nm (f).

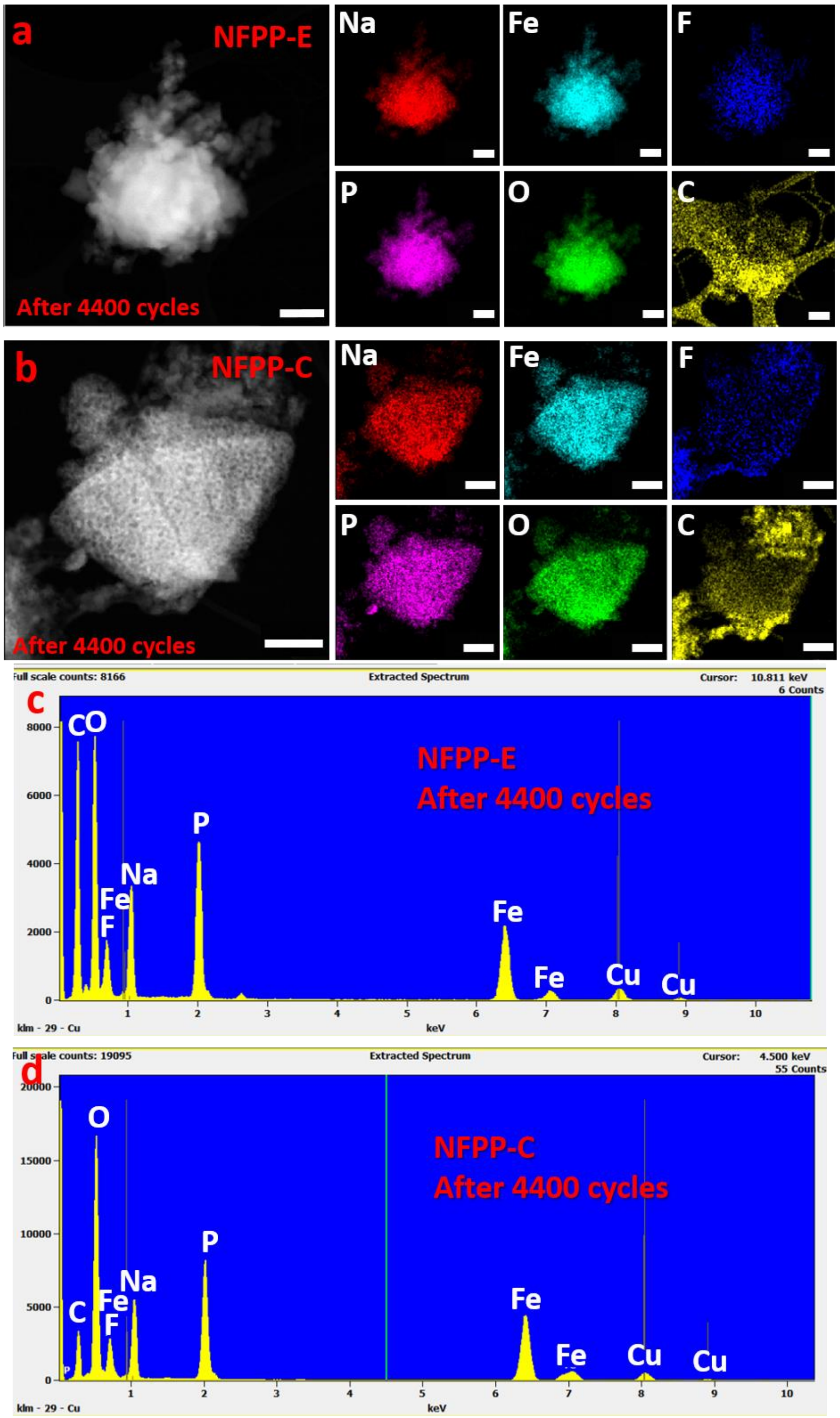


**Supplementary Figure 12** TEM images and EDS results. STEM-EDS mapping results of (a) NFPP-E and (b) NFPP-C after 4400 cycles. EDS spectra of (c) NFPP-E and (d) NFPP-C after 4400 cycles. Scale bars: 250 nm (a); 500 nm (b).

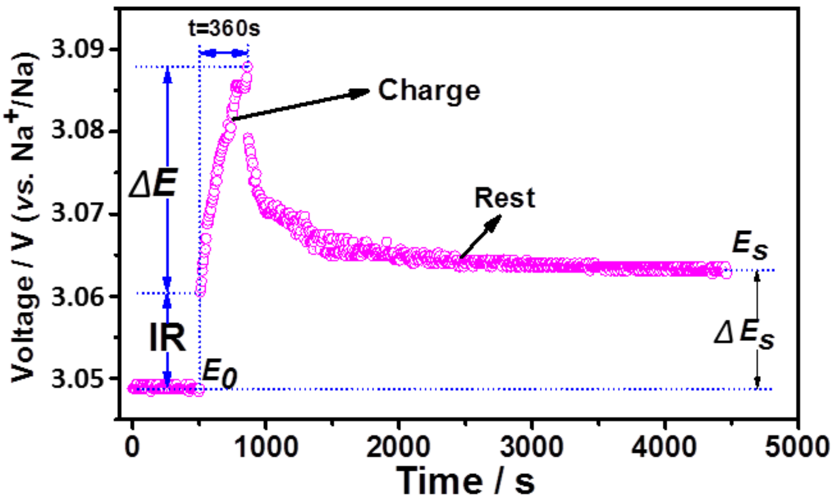


**Supplementary Figure 13** Schematic diagram of a single-step GITT experiment at ~3.07 V for NFPP-E electrode (current density: 0.05 C, 6 mA $g^{-1}$).

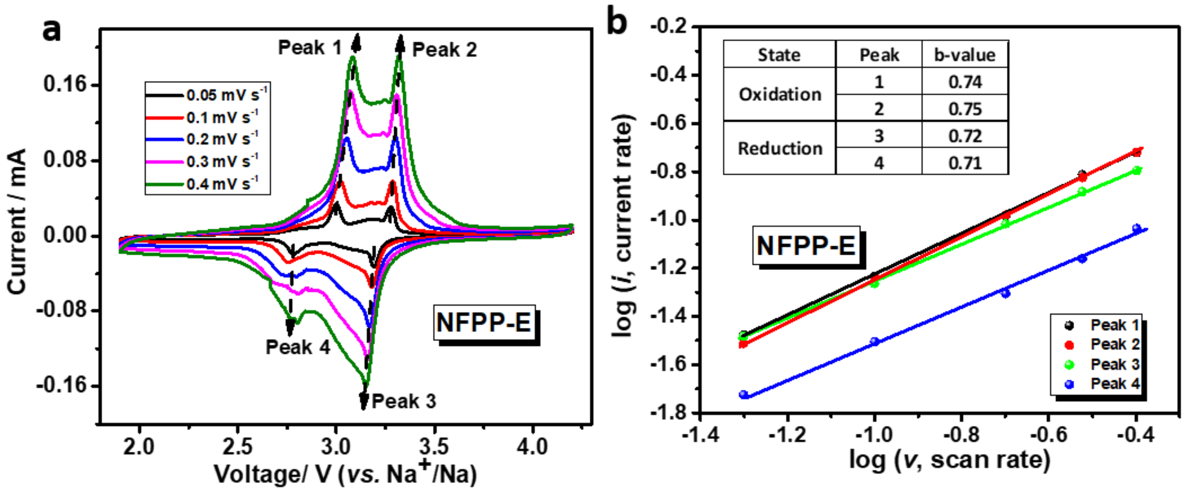


**Supplementary Figure 14** Capacitance contributions. (a) CV curves for different scan rates of NFPP-E electrode with ifour redox peaks identified. (b) The log (*i*) versus log (*v*) plots at different redox states.

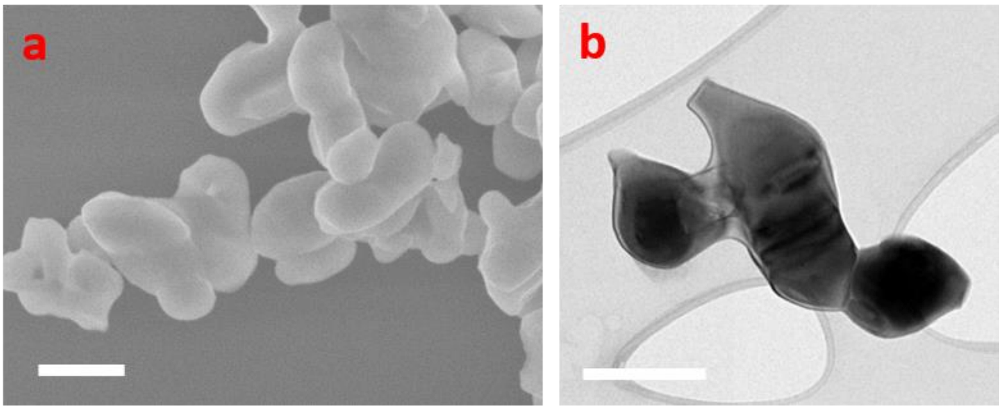


**Supplementary Figure 15** SEM and TEM images of air exposure sample. (a) SEM and (b) TEM images of the NFPP-E powder that was exposed to air for three months. Scale bars: 200 nm (a); 200 nm (b).

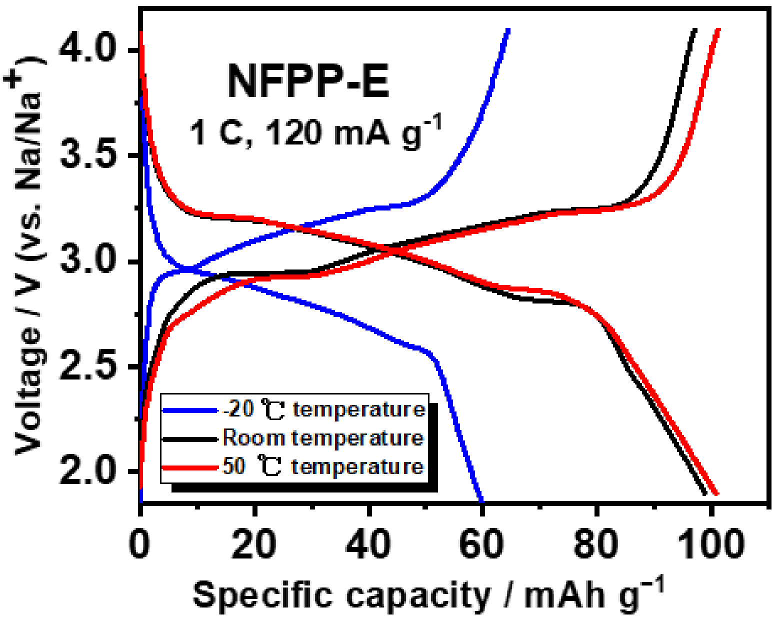


**Supplementary Figure 16** Comparison of the charge/discharge curves of NFPP-E electrodes at room temperature, 50℃ and –20 ℃, respectively.

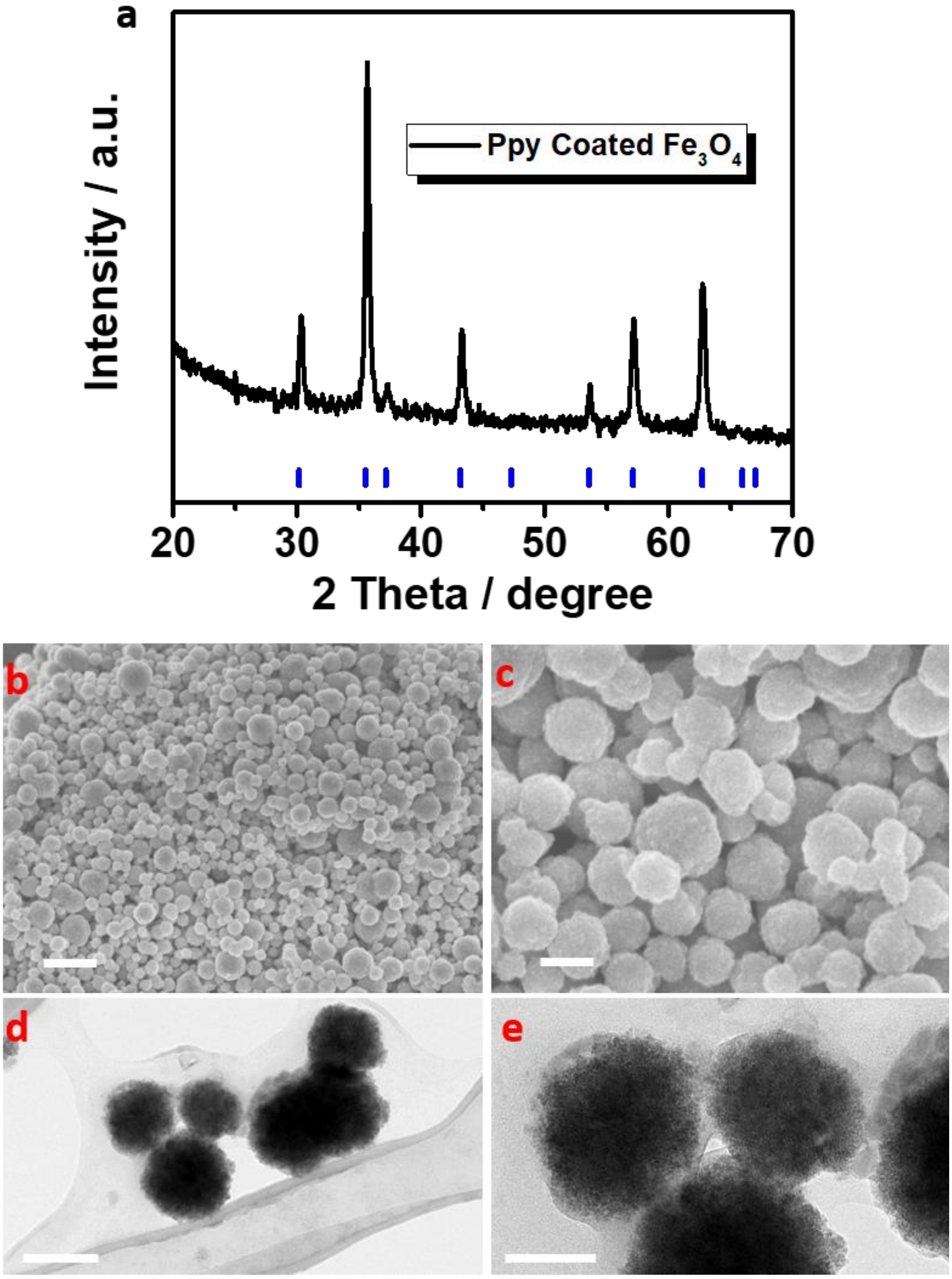


**Supplementary Figure 17** Characterization of PPy-coated $Fe_3O_4$. (a) XRD pattern of PPy-coated $Fe_3O_4$ nanospheres. The blue vertical lines are the Bragg positions extracted from $Fe_3O_4$ (PDF#88-0315). (b) and (c) SEM images, and (d) and (e) TEM images of as-obtained PPy-coated $Fe_3O_4$ nanospheres. Scale bars: 1 μm (b); 200 nm (c); 200 nm (d); 100 nm (e).

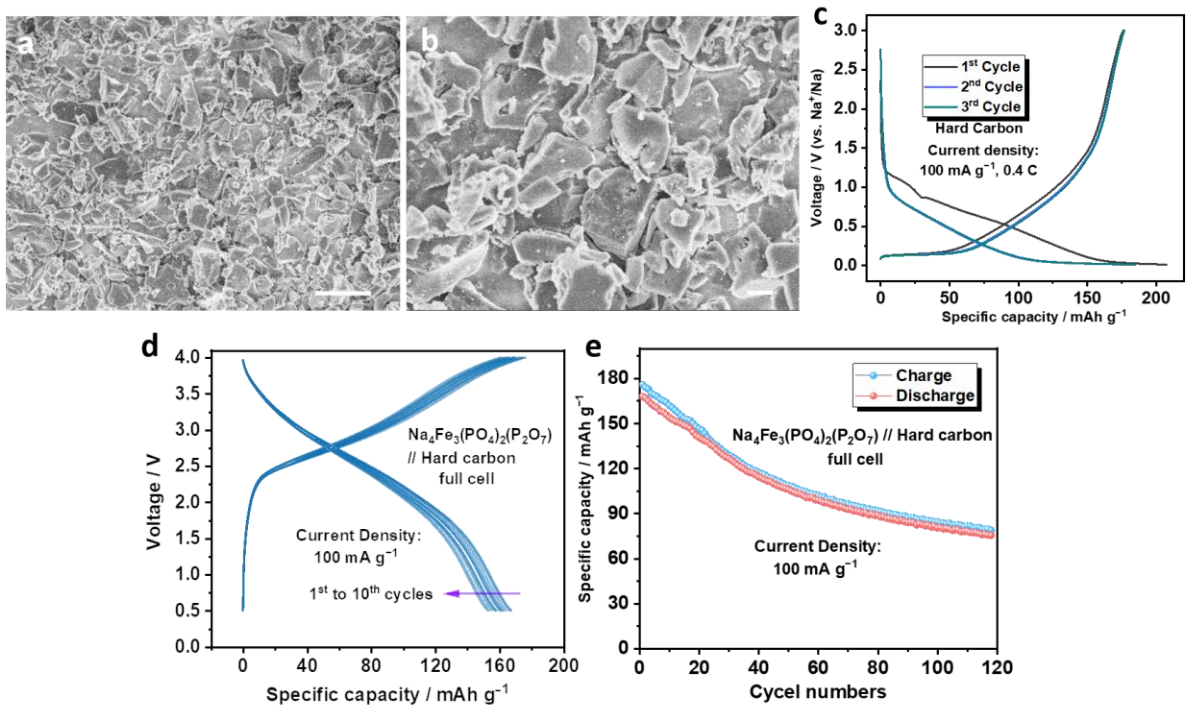


**Supplementary Figure 18** Full cell properties. (a) and (b) SEM images of purchased hard carbon. (c) Initial three cycles of purchased hard carbon (100 mA $g^{-1}$, 0.4 C). (d) Initial 10 cycles of as-prepared $Na_4Fe_3(PO_4)_2(P_2O_7)$//Hard carbon full cell within the voltage window from 0.5 V to 4.0 V at a current density of 100 mA $g^{-1}$. (e) Cyclability of as-prepared $Na_4Fe_3(PO_4)_2(P_2O_7)$//Hard carbon full cell at a current density of 100 mA $g^{-1}$. Scale bars: 10 µm (a); 2 µm (b).

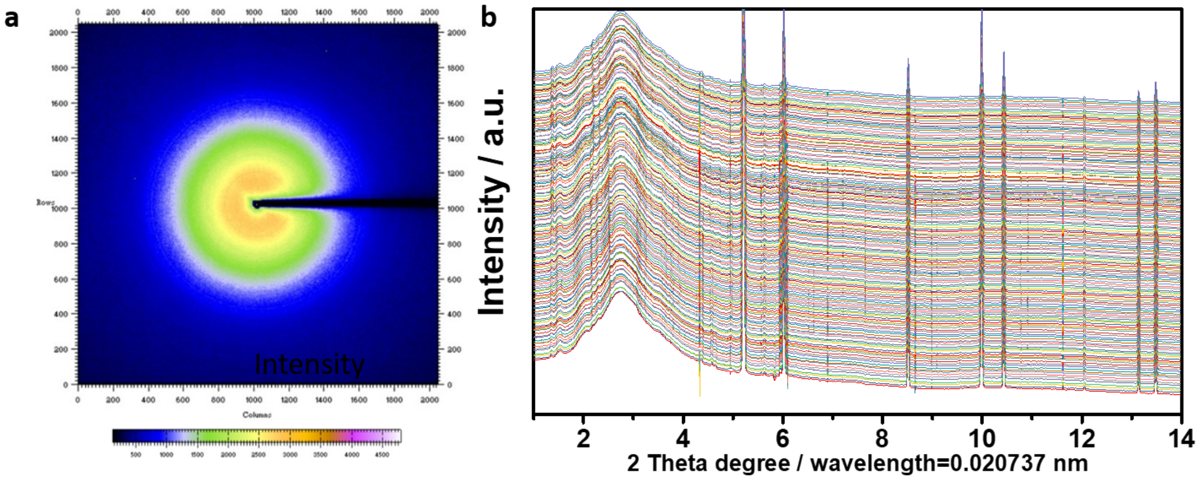


**Supplementary Figure 19** Full view of *in-situ* XRD test. (a) 2D XRD result for pristine NFPP-E electrode. (b) The whole 2$\theta$ range (wavelength = 0.020737 nm) of the as-obtained in-situ XRD patterns without the wavelength being changed to Cu $K_{\alpha}$ (0.15406 nm).

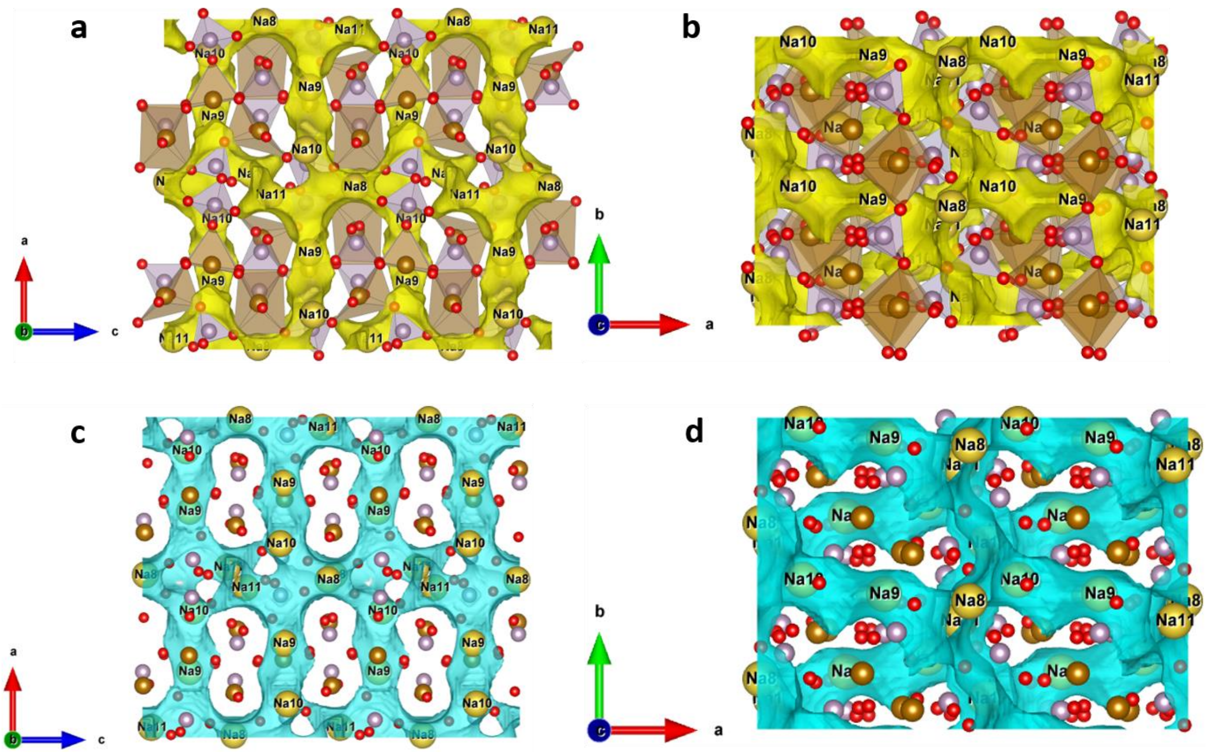


**Supplementary Figure 20** Bond-valence map. (a) and (b) Bond-valence maps of the $Na_4Fe_3(PO_4)_2P_2O_7$ material from the [010] and [001] directions, respectively. (c) and (d) Bond-valence electron voltage maps of $Na_4Fe_3(PO_4)_2P_2O_7$ material from the [010] and [001] directions, respectively.

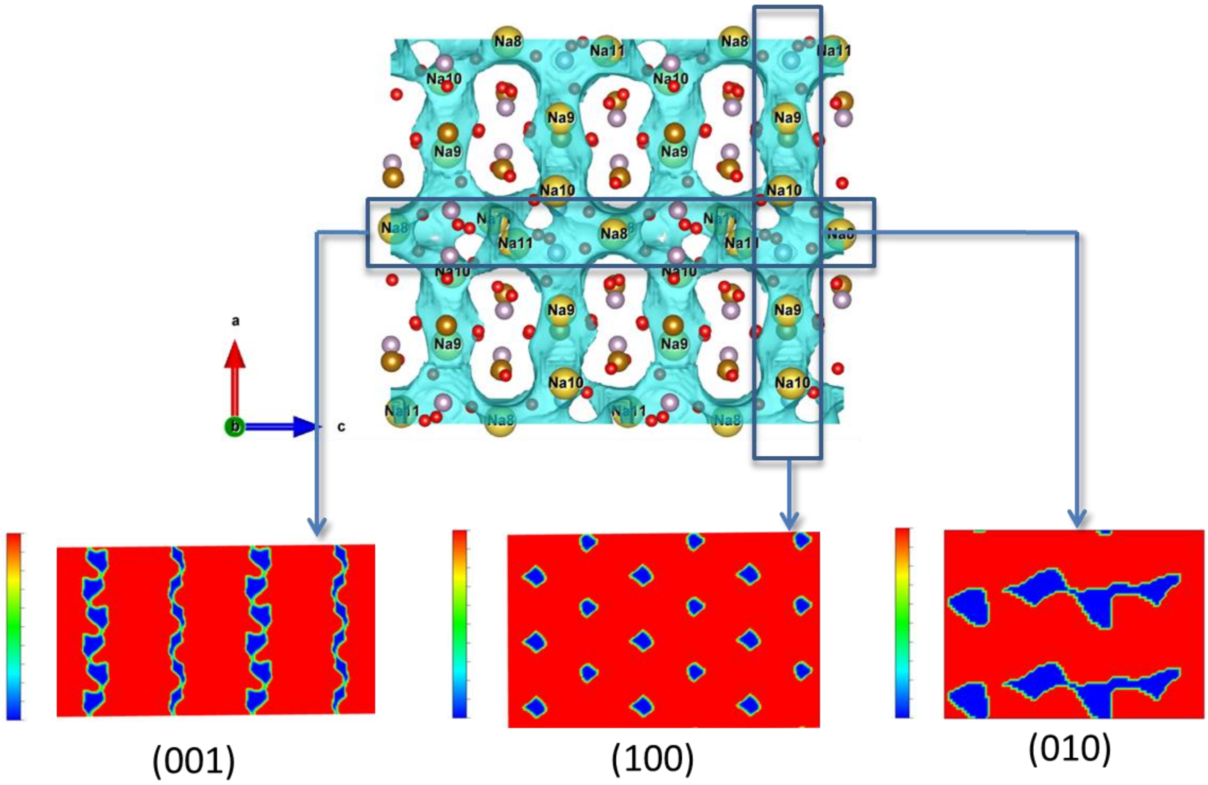


**Supplementary Figure 21** 2D slice images from different directions of the bond-valence electron voltage map.

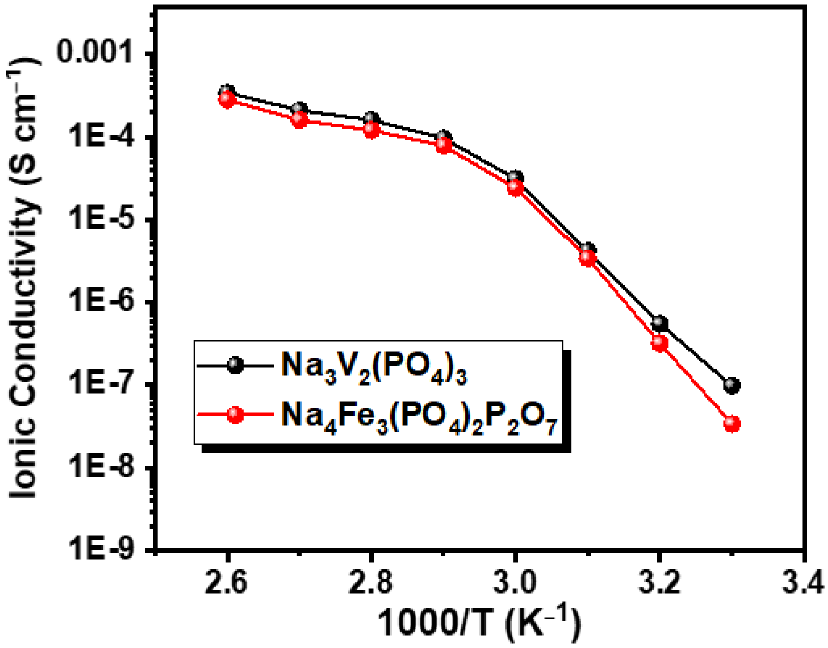


**Supplementary Figure 22** Arrhenius plots of the ionic conductivity of both $Na_3V_2(PO_4)_3$ and $Na_4Fe_3(PO_4)_2P_2O_7$ materials (Supplementary Reference 15).

**Supplementary Table 1** Detailed structural information on NFPP-E after Rietveld refinement.

| NFPP-E ($Na_4Fe_3(PO_4)_2P_2O_7$). Orthorhombic, space group *Pn2₁a* $a$ = 17.6433(6) Å, $b$ = 6.3616(4) Å, $c$ = 10.2043(4) Å, $\alpha = \beta = \gamma$ = 90°, $V$ = 1145.342(3) $Å^3$; weighted profile R-factor, $R_{wp}$: 6.97 %, square of reflection-based R factor, $R_F^2$: 12.65 %; $U_{iso}$: atomic displacement parameter. | | | | | | |
|---|---|---|---|---|---|---|
| Atom | x | y | z | frac | mult | $U_{iso}$ |
| Na1 | 0.4988(6) | 0.8819(6) | 0.9503(8) | 1.000(0) | 4 | 0.0263(7) |
| Na2 | 0.1938(2) | 0.8496(8) | 0.7254(6) | 1.000(0) | 4 | 0.0174(7) |
| Na3 | 0.4011(3) | 0.3819(6) | 0.4503(8) | 1.000(0) | 4 | 0.0147(9) |
| Na4 | 0.6606(7) | 0.6933(5) | 0.7390(6) | 1.000(0) | 4 | 0.0178(0) |
| Fe1 | 0.3374(5) | 0.1480(7) | 0.5747(6) | 1.000(0) | 4 | 0.0287(1) |
| Fe2 | 0.1645(7) | 0.6748(5) | 0.5011(1) | 1.000(0) | 4 | 0.0347(8) |
| Fe3 | 0.2500(1) | 0.3740(8) | 0.7645(5) | 1.000(0) | 4 | 0.0187(5) |
| P1 | 0.2727(1) | 0.6140(9) | 0.4682(2) | 1.000(0) | 4 | 0.0201(5) |
| P2 | 0.1272(8) | 0.1678(5) | 0.4767(7) | 1.000(0) | 4 | 0.0201(5) |
| P3 | 0.5215(6) | 0.4884(7) | 0.7108(2) | 1.000(0) | 4 | 0.0124(0) |
| P4 | 0.4863(2) | 0.1678(7) | 0.7032(7) | 1.000(0) | 4 | 0.0090(7) |
| O1 | 0.5349(8) | 0.8214(4) | 0.7379(4) | 1.000(0) | 4 | 0.0048(7) |
| O2 | 0.8650(1) | 0.3214(4) | 0.6620(5) | 1.000(0) | 4 | 0.0147(9) |
| O3 | 0.4328(0) | 0.1350(8) | 0.8120(7) | 1.000(0) | 4 | 0.0090(7) |
| O4 | 0.9328(4) | 0.1328(8) | 0.0879(2) | 1.000(0) | 4 | 0.0195(3) |
| O5 | 0.0659(3) | 0.8134(7) | 0.7449(1) | 1.000(0) | 4 | 0.0142(6) |
| O6 | 0.0762(2) | 0.4323(8) | 0.6830(4) | 1.000(0) | 4 | 0.0263(7) |
| O7 | 0.1108(6) | 0.1403(6) | 0.3236(6) | 1.000(0) | 4 | 0.0034(3) |
| O8 | 0.7064(5) | 0.5025(1) | 0.5518(4) | 1.000(0) | 4 | 0.0142(6) |
| O9 | 0.2447(7) | 0.6437(4) | 0.2447(7) | 1.000(0) | 4 | 0.0283(5) |
| O10 | 0.6108(6) | 0.1403(6) | 0.5763(3) | 1.000(0) | 4 | 0.0178(0) |
| O11 | 0.8321(5) | 0.6972(1) | 0.8321(5) | 1.000(0) | 4 | 0.0195(3) |
| O12 | 0.3321(5) | 0.6972(1) | 0.3797(6) | 1.000(0) | 4 | 0.0278(4) |
| O13 | 0.6435(9) | 0.0886(8) | 0.3357(3) | 1.000(0) | 4 | 0.0248(8) |
| O14 | 0.2064(6) | 0.5025(1) | 0.3481(5) | 1.000(0) | 4 | 0.0178(7) |
| O15 | 0.0678(4) | 0.1972(1) | 0.0202(4) | 1.000(0) | 4 | 0.0125(3) |

**Supplementary Table 2** Detailed structural information on NFPP-C after Rietveld refinement.

NFPP-C ($Na_4Fe_3(PO_4)_2P_2O_7$). Orthorhombic, space group *Pn2₁a*

$a$ = 17.6978(6) Å, $b$ = 6.3478(2) Å, $c$ = 10.2847(0) Å, α = $\beta$ = γ = 90°, $V$ = 1155.412(3) $Å^3$; weighted profile R-factor, $R_{wp}$: 7.70 %, square of reflection-based R factor, $R_F^2$: 14.87 %; $U_{iso}$: atomic displacement parameter.

| Atom | x | y | z | frac | mult | $U_{iso}$ |
|---|---|---|---|---|---|---|
| Na1 | 0.4943(6) | 0.8745(6) | 0.9456(0) | 1.000(0) | 4 | 0.0264(7) |
| Na2 | 0.1974(0) | 0.8465(8) | 0.7274(8) | 1.000(0) | 4 | 0.0164(2) |
| Na3 | 0.4134(3) | 0.3826(4) | 0.4578(0) | 1.000(0) | 4 | 0.0210(5) |
| Na4 | 0.6507(8) | 0.6943(6) | 0.7434(6) | 1.000(0) | 4 | 0.0318(4) |
| Fe1 | 0.3352(5) | 0.1478(2) | 0.5774(9) | 1.000(0) | 4 | 0.0213(7) |
| Fe2 | 0.1687(7) | 0.6703(7) | 0.5090(7) | 1.000(0) | 4 | 0.0014(3) |
| Fe3 | 0.2519(4) | 0.3746(3) | 0.7645(4) | 1.000(0) | 4 | 0.0187(5) |
| P1 | 0.2787(7) | 0.6143(9) | 0.4664(4) | 1.000(0) | 4 | 0.0101(5) |
| P2 | 0.1372(4) | 0.1663(0) | 0.4871(2) | 1.000(0) | 4 | 0.0241(4) |
| P3 | 0.5715(6) | 0.4684(7) | 0.7000(1) | 1.000(0) | 4 | 0.0134(6) |
| P4 | 0.4810(2) | 0.1601(0) | 0.7006(8) | 1.000(0) | 4 | 0.0190(4) |
| O1 | 0.5352(8) | 0.8234(6) | 0.7369(9) | 1.000(0) | 4 | 0.0148(6) |
| O2 | 0.8654(3) | 0.3264(5) | 0.6678(0) | 1.000(0) | 4 | 0.0148(9) |
| O3 | 0.4328(0) | 0.1498(8) | 0.8125(9) | 1.000(0) | 4 | 0.0194(7) |
| O4 | 0.9330(0) | 0.1326(6) | 0.0847(4) | 1.000(0) | 4 | 0.0145(4) |
| O5 | 0.0659(5) | 0.8107(7) | 0.7440(0) | 1.000(0) | 4 | 0.0142(4) |
| O6 | 0.0760(0) | 0.4410(8) | 0.6831(9) | 1.000(0) | 4 | 0.0164(3) |
| O7 | 0.1217(6) | 0.1378(6) | 0.3337(6) | 1.000(0) | 4 | 0.0134(3) |
| O8 | 0.7064(1) | 0.5025(8) | 0.5406(9) | 1.000(0) | 4 | 0.0132(4) |
| O9 | 0.2489(7) | 0.6467(4) | 0.2458(7) | 1.000(0) | 4 | 0.0273(0) |
| O10 | 0.6111(1) | 0.1473(6) | 0.5469(0) | 1.000(0) | 4 | 0.0168(0) |
| O11 | 0.8324(5) | 0.7074(0) | 0.8341(5) | 1.000(0) | 4 | 0.0190(3) |
| O12 | 0.3387(4) | 0.6964(1) | 0.3783(6) | 1.000(0) | 4 | 0.0268(4) |
| O13 | 0.6445(7) | 0.0856(8) | 0.3347(0) | 1.000(0) | 4 | 0.0268(5) |
| O14 | 0.2068(5) | 0.5036(0) | 0.3464(7) | 1.000(0) | 4 | 0.0244(7) |
| O15 | 0.0889(4) | 0.1974(9) | 0.0212(3) | 1.000(0) | 4 | 0.0142(6) |

**Supplementary Table 3** Details of the DFT calculation results.

Full cell energy ($E_{host}$) = -693.2125(2) eV    Single sodium energy ($E_{Na}$) = -1.3070(3) eV

Binding energy ($E_f$) = $E_{host}$ - （$E_{delete}$+ $E_{Na}$）

| Sodium type | $E_f$ (Binding energy) | Energy without entropy | Total energy |
|---|---|---|---|
| Na1 (A type) | -2.2395(2) eV | -689.6659(6) eV | -689.8942(1) eV |
| Na2 (A type) | -2.2400(1) eV | -689.6654(7) eV | -689.8941(2) eV |
| Na3 (A type) | -2.2393(7) eV | -689.6661(0) eV | -689.8940(9) eV |
| Na4 (A type) | -2.2392(1) eV | -689.6662(7) eV | -689.8941(1) eV |
| Na11 (B type) | -1.8774(1) eV | -690.0280(7) eV | -690.2458(2) eV |
| Na5 (B type) | -1.8954(8) eV | -690.0100(0) eV | -690.2455(3) eV |
| Na7 (B type) | -1.8968(6) eV | -690.0086(2) eV | -690.2453(6) eV |
| Na9 (B type) | -1.8755(6) eV | -690.0298(9) eV | -690.2458(6) eV |
| Na10 (B type) | -1.8762(5) eV | -690.0292(3) eV | -690.2458(4) eV |
| Na6 (B type) | -1.8963(1) eV | -690.0091(7) eV | -690.2455(6) eV |
| Na8 (B type) | -1.8968(2) eV | -690.0086(6) eV | -690.2454(2) eV |
| Na12 (B type) | -1.8795(6) eV | -690.0259(2) eV | -690.2456(4) eV |
| Na13 (C type) | -2.2792(7) eV | -689.6262(1) eV | -689.8405(0) eV |
| Na14 (C type) | -2.2807(2) eV | -689.6247(6) eV | -689.8396(5) eV |
| Na15 (C type) | -2.2793(3) eV | -689.6261(4) eV | -689.8407(2) eV |
| Na16 (C type) | -2.2794(0) eV | -689.6260(7) eV | -689.8406(6) eV |

## Supplementary Notes

**Supplementary Note 1** | As shown in Supplementary Figure 13, the steady-state voltage $\Delta E_s$ can be obtained from the *D*-value for the diffusion coefficient between the last round stable voltage ($E_0$) and the current stable voltage ($E_s$). $\Delta E_\tau$ is obtained *via* a 360 second charging process. The process for sodium ion diffusion is assumed to follow Fick's second law of diffusion. With a series of parameter simplifications, for sufficient rest time ($\tau \ll L^2 / D_{Na^+}$), the equation for $D_{Na^+}$ can be considered as:

$$D_{Na+} = \frac{4L^2}{\pi\tau}\left(\frac{\Delta E_s}{\Delta E_\tau}\right)^2 \qquad (\tau \ll L^2 / D_{Na+}). \qquad \text{(Supplementary Equation 1)}$$

Where $L$ is the thickness of the coated material on the electrode, $\tau$ is the charging time per round, and *л* is pi (considered as 3.1416). Based on Supplementary Equation 1, the chemical diffusion coefficient $D_{Na^+}$ can be calculated as a function of the charging/discharging voltage in the cell.

**Supplementary Note 2 |** The effect of the capacitance in Supplementary Figure 14 of the battery reaction system can be measured according to the following Supplementary Equation Equations 2 and Supplementary Equation 3:

$$i = a\, v^{b} \qquad \text{(Supplementary Equation 2)}$$

$$\log i = b \times \log v + \log a \qquad \text{(Supplementary Equation 3)}$$

where $i$ is the current density, $v$ is the sweep rate, and $a$ and $b$ are adjustable parameters. If the $b$-value approaches or is above 1, the electrochemical reaction system is mainly controlled *via* capacitance, while if the $b$-value is around 0.5, the insertion/de-insertion process for $Na^+$ ions dominates the system.

The over-all pseudocapacitance contribution from 1.5-4.0 V can be calculated according to Supplementary Equation Equation 3:

$$i(V) = k_1\, v + k_2\, v^{0.5} \qquad \text{(Supplementary Equation 4)}$$

where $v$ is the sweep rate, and $V$ is the fixed potential. By determining both the parameters $k_1$ and $k_2$, it is thus possible to determine the capacitive contribution to the total current.

**Supplementary References:**


1. Fang, Y. *et al*. High-performance olivine $NaFePO_4$ microsphere cathode synthesized by aqueous electrochemical displacement method for sodium-ion batteries. *ACS Appl. Mater. Interfaces* **7**, 17977-17984 (2015).
2. Rajagopalan, R. *et al*. Improved reversibility of $Fe^{3+}/Fe^{4+}$ redox couple in sodium super ion conductor type $Na_3Fe_2(PO_4)_3$ for sodium-ion batteries. *Adv. Mater.*, **29**, 1605694 (2017).
3. Liu, D. & Palmore G. T. R. Synthesis, crystal structure, and electrochemical properties of alluaudite $Na_{1.702}Fe_3(PO_4)_3$ as a sodium-ion battery cathode. *ACS Sustainable Chem. Eng.*, **5**, 5766-5771 (2017).
4. Smiley, D. L. & Goward, G. R. Ex situ $^{23}Na$ solid-state NMR reveals the local Na-ion distribution in carbon-coated $Na_2FePO_4F$ during electrochemical cycling. *Chem. Mater.*, **28**, 7645-7656 (2016).
5. Lin, B., Zhang, S. & Deng, C. Understanding the effect of depressing surface moisture sensitivity on promoting sodium intercalation in coral-like $Na_{3.12}Fe_{2.44}(P_2O_7)_2$/C synthesized *via* a flash-combustion strategy. *J. Mater. Chem. A*, **4**, 2550-2559 (2016).
6. Chen, M. *et al*. Carbon-coated $Na_{3.32}Fe_{2.34}(P_2O_7)_2$ cathode material for high-rate and long-life sodium-ion batteries. *Adv. Mater.*, **29**, 1605535 (2017).
7. Barpanda, P. *et al*. $Na_2FeP_2O_7$: a safe cathode for rechargeable sodium-ion batteries. *Chem. Mater.*, **25**, 3480-3487 (2013).
8. Kim, H. *et al*. Understanding the electrochemical mechanism of the new iron-based mixed-phosphate $Na_4Fe_3(PO_4)_2(P_2O_7)$ in a Na rechargeable battery. *Chem. Mater.*, **25**, 3614-3622 (2013).
9. Guan, W. *et al*. A high capacity, good safety and low cost $Na_2FeSiO_4$-based cathode for rechargeable sodium-ion battery. *ACS Appl. Mater. Interfaces*, **9**, 22369-22377 (2017).
10. Dwibedi, D. *et al*. Ionothermal synthesis of high-voltage alluaudite $Na_{2+2x}Fe_{2-x}(SO_4)_3$ sodium insertion compound: structural, electronic, and magnetic insights. *ACS Appl. Mater. Interfaces*, **8**, 6982-6991 (2016).
11. Barpanda, P., Oyama, G., Nishimura, S., Chung, S. C. & Yamada A. A 3.8-V earth-abundant sodium battery electrode. *Nat. Commun.*, **5**, 4358 (2014).
12. Goñi, A., Iturrondobeitia, A., Gil de Muro, I., Lezama, L. & Rojo,T. $Na_{2.5}Fe_{1.75}(SO_4)_3$/Ketjen/rGO: an advanced cathode composite for sodium ion batteries. *J Power Sources*, **369**, 95-102 (2017).
13. Yao, W *et al*. Reinvestigation of $Na_2Fe_2(C_2O_4)_3\cdot 2H_2O$: an iron-based positive electrode for secondary batteries. *Chem. Mater.*, **29**, 9095-9101 (2017).
14. Lu, J., Nishimura, S.-i. & Yamada, A. Polyanionic solid-solution cathodes for rechargeable batteries. *Chem. Mater.*, **29**, 3597-3602 (2017).
15. Gao, R. *et al*. Nanofiber networks of $Na_3V_2(PO_4)_3$ as a cathode material for high performance all-solid-state sodium-ion batteries. *J Mater. Chem. A*, **5**, 5273-5277 (2017).
